# Multimodal Approach for Recalcitrant Melasma Using Picosecond Laser and Topical JAK Inhibition: A Case Report

**DOI:** 10.1111/jocd.70783

**Published:** 2026-03-25

**Authors:** Hasan Ashkanani, Maryam AlZaabi, Abdulaziz AlRasheed, Wael AlDaraji

**Affiliations:** ^1^ Department of Dermatology Al‐Amiri Hospital Kuwait City Kuwait; ^2^ Department of Dermatology Mubarak Al‐Kabeer Hospital Jabriya Kuwait; ^3^ Department of Plastic Surgery Kuwait Hospital Sabah AlSalem Kuwait; ^4^ Dr. Gera Tagani Research Centre, Newcastle University London UK

**Keywords:** hyper‐pigmentation, LASER, Melasma

## Abstract

**Background:**

Melasma is a chronic hyperpigmentation disorder with high relapse rates, particularly in Fitzpatrick skin types III–V. Increasing evidence implicates cytokine‐driven inflammation and visible light–induced melanogenesis as key contributors to disease persistence. These mechanisms support the rationale for exploring a multimodal therapeutic approach.

**Case Presentation:**

A woman in her early 30s with phototype IV presented with a 5‐year history of recalcitrant centrofacial melasma. Previous treatments—including hydroquinone, oral tranexamic acid, and superficial chemical peels—produced only transient and incomplete improvement. Baseline Melasma Severity Index (MSI) score was 7.9, consistent with moderate‐to‐severe disease.

**Intervention:**

Treatment consisted of low‐fluence picosecond 755 nm alexandrite laser, short‐term topical corticosteroid post‐procedure, initiation of topical tofacitinib 2% cream twice daily for 11 months, and continuous iron oxide–based visible light–blocking photoprotection. Laboratory monitoring during therapy remained normal.

**Outcome:**

At 11 months, the patient achieved near‐total clearance with an MSI reduction from 7.9 to 0.10 (98.7% improvement). No adverse events, post‐inflammatory hyperpigmentation, or relapse were observed, including 3 months after treatment cessation.

**Conclusion:**

This case illustrates the potential clinical value of combining pigment‐targeted picosecond laser therapy with topical JAK inhibition and visible light–specific photoprotection for recalcitrant melasma. The durable remission achieved supports further investigation into anti‐inflammatory and photoreactive pathway modulation as adjunctive strategies in melasma management.

## 1 Introduction

Melasma is a chronic, relapsing hyperpigmentary disorder characterized by aberrant melanogenesis, with contributory roles from hormonal, inflammatory, and photobiological pathways. The condition poses a particular therapeutic challenge in Fitzpatrick skin types III–V, where the risk of post‐inflammatory hyperpigmentation (PIH) and limited response to conventional therapies often leads to patient frustration and frequent relapse.

Recent studies have expanded our understanding of melasma pathogenesis beyond ultraviolet (UV) exposure, implicating visible light (VL) as a potent melanogenic stimulus. VL acts through opsin‐3 receptor signaling and oxidative stress generation, particularly in melanocompetent skin types, compounding pigmentation persistence in darker phototypes as demonstrated by Mahmoud et al. [1]. Simultaneously, cutaneous inflammation, driven by keratinocyte‐derived cytokines (IL‐6, IL‐1β, TNF‐α), is now recognized as a critical upstream modulator of melanocyte activity, suggesting an expanded therapeutic role for immunomodulation. There is an unmet need for protocols targeting both inflammatory and photoreactive elements of melasma.

JAK/STAT pathway inhibitors, such as topical tofacitinib, have emerged as a potential area of investigation in pigmentary disorders by modulating cytokine‐mediated keratinocyte–melanocyte signaling and blocking IFN‐γ and IL‐6‐mediated keratinocyte–melanocyte cross‐talk, thereby suppressing cytokine‐driven melanogenesis. Although off‐label, their potential in pigmentary disorders is supported by preliminary studies in vitiligo and post‐inflammatory hyperpigmentation [2, 3].

In this report, we present a case of facial melasma in a phototype IV patient successfully treated with picosecond laser, topical JAK inhibition, and visible light–blocking sunscreen. To our knowledge, this represents one of the earliest documented uses of topical tofacitinib 2% cream in combination with laser therapy for melasma.

## 2 Case Presentation

A woman in her early 30s with Fitzpatrick phototype IV presented with a 5‐year history of persistent, symmetrical centrofacial melasma. Prior treatments included 4% hydroquinone, oral tranexamic acid (250 mg BID for 12 weeks), and monthly superficial glycolic acid peels, which yielded only temporary improvement followed by relapse. She denied hormonal therapy use, systemic illness, or family history of pigmentary disorders.

**FIGURE 1–2:**
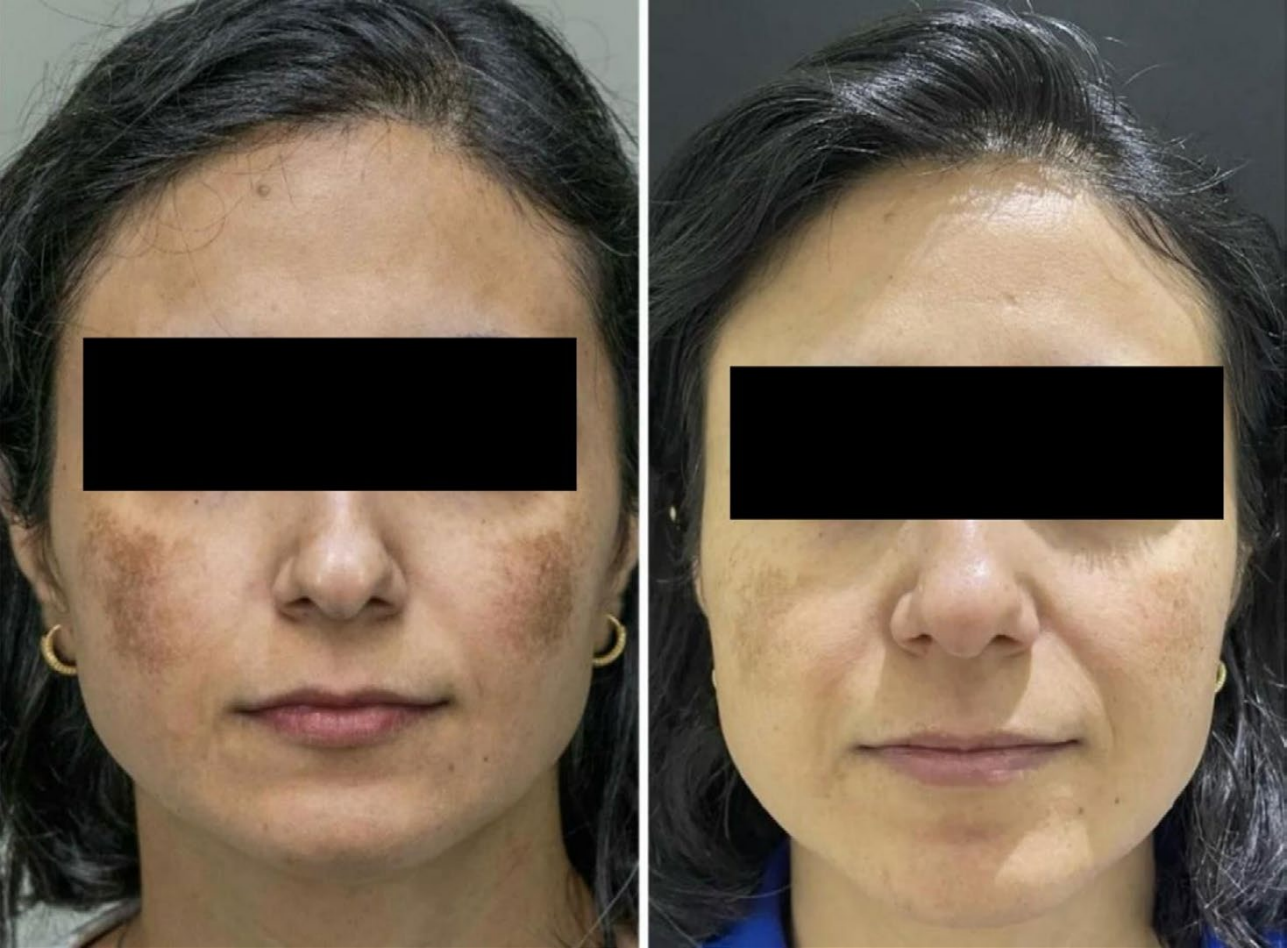
Clinical progression of melasma treated with 755 nm picosecond alexandrite laser, short‐term topical corticosteroid, long‐term topical tofacitinib 2% cream, and tinted visible light‐blocking sunscreen (Photographs arranged left to right). Baseline presentation: Dense, patchy hyperpigmentation affecting bilateral malar and zygomatic areas. Post‐treatment (11 months): Marked clearance of pigmentation and restoration of even skin tone.

On examination, patchy hyperpigmentation was noted over the malar, zygomatic, nasal bridge, and upper lip areas, with ill‐defined borders. Wood's lamp accentuation supported a mixed epidermal‐dermal pattern. The baseline Melasma Severity Index (MSI) score was 7.9, indicating moderate‐to‐severe melasma (Figure 1). She underwent a multimodal regimen consisting of:

- Picosecond 755 nm alexandrite laser (PicoSure Pro, Cynosure/Hologic, Westford, MA, USA).
  ○ Flat lens: 10 mm, 0.25 J/cm^2^, 5 Hz.
  ○ Focus lens: 6 mm, 0.7 J/cm^2^, 2.5 Hz.
  ○ Sessions performed under topical anesthesia and following cooling.

Anti‐inflammatory: Fucicort (Leo Pharma, Ballerup, Denmark), containing fusidic acid 2% and hydrocortisone acetate 1%, applied twice daily for 3 days post‐laser.

Topical JAK inhibition: Compounded topical tofacitinib 2% cream (custom compounded in a lipophilic cream base by a licensed compounding pharmacy, Baghdad, Iraq) was initiated 48 h post‐laser and applied twice daily, started 48 h post‐laser, twice daily for 11 months.

Photoprotection: Heliocare 360° (Cantabria Labs, Madrid, Spain), an iron oxide–containing tinted sunscreen, applied twice daily [4].

Follow‐up visits were scheduled at 1, 3, 6, and 11 months. No systemic therapy was given. Laboratory monitoring (CBC, liver enzymes, and creatinine) at baseline, 3, and 6 months was unremarkable, and no adverse effects related to tofacitinib were reported.

At final follow‐up (month 11), the patient exhibited near‐total resolution of melasma with homogeneous pigmentation, smoother texture, and an estimated MSI score reduction from 7.9 to 0.10 (an 98.7% reduction) (Figure 2). No relapse or adverse events were noted, and the patient remained off treatment at last contact 3 months after cessation.

## 3 Discussion

This case illustrates the potential of a rational, multimodal approach to treating recalcitrant melasma in a patient with phototype IV skin.

### 3.1 Laser Selection for Pigment Disruption

Picosecond 755 nm alexandrite laser. offer shorter pulse duration (450–750 ps), generating photoacoustic effects with minimal thermal injury, making them ideal for melanin targets in darker skin without triggering inflammation‐driven rebound [5]. Compared to Q‐switched nanosecond lasers, picosecond devices offer superior efficacy and safety in darker phototypes, with reduced risk of PIH [5]. In our case, a conservative fluence protocol was used, and no pigmentary complications occurred.

### 3.2 JAK Inhibition for Inflammation Modulation

Recent insights implicate cytokine‐driven keratinocyte–melanocyte cross‐talk, particularly via IFN‐γ and IL‐6, in sustaining melanogenesis in melasma [1]. JAK inhibitors interrupt this axis by blocking downstream STAT activation. Although topical JAK inhibition remains off‐label for melasma, pilot studies and case reports support its utility in pigmentary disorders such as post‐inflammatory hyperpigmentation and vitiligo [3]. In a Phase 3 trial, ruxolitinib 1.5% cream demonstrated significant repigmentation in vitiligo, with a favorable safety profile over 24 weeks [3]. Additionally, small‐scale reports of tofacitinib and ruxolitinib use in PIH have shown promising outcomes, though data remain preliminary.

The patient tolerated 11 months of compounded topical tofacitinib 2% cream without laboratory abnormalities or adverse effects. However, given the lack of formal pharmacokinetic data on systemic absorption from long‐term topical use, safety surveillance and regulatory caution are warranted. As of this writing, neither topical tofacitinib nor ruxolitinib is FDA‐approved for melasma or hyperpigmentation.

### 3.3 Visible Light Protection: A Neglected Pillar

Visible light contributes to melasma exacerbation by activating opsin 3 and inducing oxidative stress. A randomized controlled trial demonstrated that VL‐blocking sunscreen significantly outperforms UV‐only formulations in reducing pigmentation recurrence. Only iron oxide–based tinted sunscreens provide effective VL shielding [6].

In summary, the patient's outcome supports the hypothesis that long‐term suppression of inflammatory and photoreactive triggers, alongside pigment‐targeted laser therapy, can induce durable clearance in melasma. This case report is hypothesis‐generating in nature and does not establish efficacy or causality.

## 4 Conclusion

This case highlights the successful use of a mechanism‐based, multimodal regimen for the treatment of recalcitrant melasma in a phototype IV patient. By addressing the core pathogenic drivers, which are melanin overproduction, cytokine‐driven inflammation, and visible light–induced photoreactivation, long‐term remission was achieved without adverse effects.

The combination of low‐fluence picosecond 755 nm alexandrite laser, a compounded topical JAK inhibitor (tofacitinib 2% cream), and iron oxide–based VL‐blocking sunscreen led to sustained pigment clearance and high patient satisfaction. While the safety profile was favorable in this case, the off‐label use of topical JAK inhibitors underscores the need for further investigation into their pharmacokinetics, systemic absorption, and long‐term efficacy in pigmentary disorders.

This report adds to the body of literature supporting an approach to melasma, particularly in patients with darker skin types, where relapse rates are higher. Controlled studies are warranted to validate these findings and to develop treatment protocols incorporating targeted immunomodulators and VL‐specific photoprotection.

## Funding

The authors have nothing to report.

## Ethics Statement

Approved by Health IRB.

## Consent

The authors obtained written consent from participants for their information to be published in print and online, and with the understanding that this information and photographs may be publicly available. The participant consent form was not provided to the journal but is retained by the authors.

## Conflicts of Interest

The authors declare no conflicts of interest.

## Data Availability

The data that support the findings of this study are available on request from the corresponding author. The data are not publicly available due to privacy or ethical restrictions.
